# The Impact of Acute Exercise on Blood Work Parameters: A Case Report of a Healthy Male University Student

**DOI:** 10.7759/cureus.70018

**Published:** 2024-09-23

**Authors:** Mina Al Akko, Miray Maher, Parisa Airia

**Affiliations:** 1 Internal Medicine, Temerty Faculty of Medicine, University of Toronto, Toronto, CAN; 2 Family and Community Medicine, Temerty Faculty of Medicine, University of Toronto, Toronto, CAN

**Keywords:** acute exercise, alt : alanine transaminase, ast (aspartate aminotransferase), bilirubin, creatine kinase

## Abstract

This case report presents a 22-year-old male who exhibited critically high levels of creatine kinase (CK) (3,600 U/L), elevated liver transaminases (alanine aminotransferase (ALT) 90 U/L; aspartate aminotransferase (AST) 54 U/L), and elevated total bilirubin (30 µmol/L) following vigorous physical activity. The patient’s blood work parameters normalized after a short period of exercise abstinence, highlighting the impact of acute exercise on blood work parameters and the importance of considering recent physical activity when interpreting laboratory results.

## Introduction

Acute physical activity is known to elicit various physiological and chemical responses, one of which is its impact on blood work parameters. Specifically, a well-established phenomenon in the medical field is the increase in creatine kinase (CK) levels in response to exercise. This finding should not be surprising, as CK is an enzyme found in skeletal muscle, brain, and heart cells. When muscle cells experience micro-tearing due to physical stress, this enzyme leaks into the bloodstream, resulting in an increased concentration when tested [1].

What is less known, however, is that CK is not the only enzyme that can be altered by exercise. What are traditionally known as liver enzymes, alanine aminotransferase (ALT) and aspartate aminotransferase (AST), can also be affected. Although both enzymes are associated with liver function, they are also present in skeletal muscle cells. Similar to CK, post-exercise micro-tearing can cause these enzymes to leak into the bloodstream, leading to a transient increase in their concentrations. Additionally, exercise has a temporary, direct impact on liver cells and blood flow to the liver, which could further augment the alterations in ALT and AST levels, as will be described shortly [2].

This case report aims to highlight the alteration in blood work parameters in response to acute, moderate-to-high intensity exercise in a healthy male university student, and the importance of considering recent physical activity when interpreting laboratory results to avoid misinterpretation, misdiagnosis, and heightened patient anxiety.

## Case presentation

A 22-year-old male presented to his family doctor’s clinic for an initial introductory visit with no concerns. On physical examination, the patient appeared alert, was not in acute distress, and was speaking in full sentences. His cardiac examination revealed normal heart sounds (S1 and S2) without any murmurs. The respiratory examination showed normal lung sounds bilaterally, without adventitious sounds. The abdominal examination was unremarkable, with no stigmata of liver disease or hepatomegaly. The rest of the examination, including the cranial nerve assessment, was also unremarkable.

The patient's medical history was notable only for a deviated septum, for which septoplasty had been performed. He had no known environmental or drug allergies. His family history was significant for hypertension and diabetes.

The primary care provider decided to request bloodwork testing to obtain a baseline for future reference. The results showed critically high levels of CK and elevated ALT, AST, and total bilirubin (Table 1). While the elevated CK was expected to be related to exercise, the elevated ALT, AST, and bilirubin were not typically associated with it. This prompted an initial consideration of fatty liver disease.

**Table 1:** Laboratory values upon presentation one day after vigorous exercise and repeat test results after two days of complete exercise abstinence. CK: Creatine kinase; ALT: Alanine transaminase; AST: Aspartate transaminase.

	CK (U/L)	ALT (U/L)	AST (U/L)	Total Bilirubin (µmol/L)
Reference ranges	49-397	17-63	8-33	5-21
Initial test results	3600	90	54	30
Repeat test results	100	39	19	12

Upon further inquiry, the patient informed his primary care provider that he had engaged in vigorous physical activity (a total of 3 hours of intense weightlifting and endurance training) the day before his blood sample was obtained. Consequently, it was agreed to repeat the blood tests after two days of complete exercise abstinence before proceeding with additional workup. The repeat tests showed a reduction in CK, ALT, AST, and total bilirubin to normal levels (Table 1).

## Discussion

Effect of exercise on CK

CK, an enzyme found in skeletal muscle, brain, and heart, plays a pivotal role in the production of adenosine triphosphate (ATP), the primary energy currency of the cell [3]. It catalyzes the conversion of creatine and ATP to creatine phosphate and adenosine diphosphate (ADP). Creatine phosphate serves as the primary store for high-energy phosphates, which can be transferred to ADP to create ATP when needed, such as during physical activity [3]. Following acute exercise, particularly exercise that involves eccentric (lengthening) muscle contraction, CK levels in the blood increase [4]. This occurs, as previously described, due to the leakage of the enzyme into the bloodstream after cellular damage and micro-tearing induced by physical exercise [4]. Understanding this physiological basis is critical in interpreting elevated CK levels in patients within the context of physical activity.

Effect of exercise on bilirubin

Exercise can also transiently increase bilirubin levels, a by-product of hemoglobin and myoglobin metabolism. Physical stress on muscle cells leads to the breakdown of myoglobin, a heme-containing compound in muscle cells used for oxygen storage and release [5]. When muscle cells are damaged, myoglobin is broken down, contributing to elevated bilirubin levels [5]. In addition, exercise-induced hemolysis contributes to rising bilirubin levels through red blood cell injury and rupture, releasing free hemoglobin, which is consequently metabolized into bilirubin [6]. Moreover, exercise can temporarily decrease blood flow to the liver, further contributing to transient hyperbilirubinemia, as blood is shunted to more metabolically demanding tissues such as muscles [7]. Reduced blood flow to the liver may transiently decrease the uptake, conjugation, and excretion of bilirubin [8]. Therefore, exercise can cause transient unconjugated hyperbilirubinemia due to myoglobin and hemoglobin breakdown, with a small additional contribution of conjugated hyperbilirubinemia due to its impact on liver blood flow.

Effect of exercise on liver transaminases

ALT and AST are enzymes typically associated with liver function as they play a critical role in amino acid metabolism. ALT is primarily found in the liver, where it facilitates the conversion of alanine and α-ketoglutarate to pyruvate and glutamate, which are vital steps in gluconeogenesis and amino acid metabolism. AST, while also present in the liver, is more widely distributed, being found in the heart, skeletal muscles, kidneys, brain, and red blood cells [9]. It catalyzes the transfer of an amino group from aspartate to α-ketoglutarate, forming oxaloacetate and glutamate [9]. Since these enzymes are present in muscle cells, their serum levels can rise in response to muscle damage via a similar mechanism to CK leakage [2], in addition to the impact on liver cells and blood delivery described earlier. Transaminase levels can remain elevated for up to seven days post-exercise, and the magnitude of the elevation is proportional to exercise intensity and duration [10].

Effect of exercise on hematological parameters

Acute exercise can also transiently increase RBCs through many mechanisms. One key factor is stimulating erythropoiesis, the process of producing new RBCs in the bone marrow. This occurs in response to decreased blood flow to the kidney (as the blood is shunted to more metabolically active tissue) and the “pseudo” hypoxia that results. This signal is detected by the kidneys, which then secrete erythropoietin to promote the production of RBCs [11]. Moreover, exercise can promote the mobilization of more RBCs from the spleen, thereby increasing the total “apparent” RBC count if blood were to be drawn shortly after exercise [12]. This is followed by a decrease in RBCs due to hemolysis (which contributes to the rise in bilirubin), although this effect is trivial and rarely reflects as true erythrocytopenia [6]. Similarly, white blood cells (WBCs) also increase slightly post-exercise due to the systemic immune response to physiological stress induced by exercise from the micro-tearing of muscle fibers, leading to the mobilization of WBCs into the muscle to promote tissue healing and regeneration [13]. The release of cortisol during exercise (and its prolonged action post-exercise) may further modulate the WBC count by affecting the release of inflammatory molecules and vascular permeability [14].

Effect of exercise on glucose and lipid metabolism

When it comes to bioenergetics, exercise has a profound impact on glucose levels. It promotes non-insulin dependent uptake of glucose by skeletal muscle cells by facilitating the translocation of glucose transporter type 4 (GLUT4) to the cell surface, which allows glucose to enter the cells without insulin signaling [15]. Moreover, exercise can enhance the cells’ sensitivity to insulin, further enhancing glucose uptake and glycogen storage [15]. This results in a transient decrease in glucose levels immediately post-exercise.

Lipid metabolism may also be altered in response to acute exercise. During exercise, there is an immediate increase in cholesterol and high-density lipoprotein (HDL) levels [16]. This transient rise can be attributed to the mobilization of lipid stores to meet the metabolic demands in response to physical stress and the de novo synthesis of steroid hormones [17]. Low-density lipoprotein (LDL) may decrease slightly immediately after exercise due to increased uptake of LDL by tissues requiring cholesterol for tissue repair and biosynthesis [16]. Triglycerides tend to decrease for several days post-exercise due to lipolysis, the process of breaking down triglycerides into fatty acids and glycerol. This process occurs primarily when the body shifts its metabolic demands to fat metabolism (typically after glycogen stores have been depleted) [18].

Effect of exercise on other metabolites

Creatinine and urea levels are also affected by exercise. Both rise for days post-exercise due to muscle protein breakdown [19,20]. Creatinine, a waste product of muscle metabolism, accumulates as muscle tissue undergoes repair and hypertrophy [20]. Similarly, urea, a byproduct of protein catabolism, increases as the body metabolizes micro-torn muscle proteins via the urea cycle [19].

Clinical implications

Exercise can cause transient changes in various blood components, including elevated levels of CK, ALT, AST, mixed unconjugated/conjugated hyperbilirubinemia, glucose and lipid metabolism, and kidney function parameters. To mitigate this issue, it might be beneficial to advise patients to avoid physical activities prior to undergoing blood tests. Additionally, it is extremely valuable to obtain a comprehensive exercise history from the patient, especially in young, athletic patient demographics. This history should include the type of exercise, duration of each session, frequency of workouts per week, intensity level, and potential supplement use. It might also be beneficial to measure CK, which is not commonly ordered in routine tests, in the right demographics if exercise-induced changes in bloodwork parameters are suspected, providing a marker that increases the index of suspicion for exercise-induced changes.

## Conclusions

This case report highlights the impact of acute, moderate-to-high intensity exercise on various blood work parameters, including CK, liver transaminases, and total bilirubin. Patients with abnormal laboratory results should be advised to observe a period of exercise abstinence to distinguish between true pathology and exercise-mediated changes. Clinicians should obtain a detailed exercise history to avoid misinterpretation of laboratory tests, misdiagnoses, and unwarranted patient anxiety, thereby improving the accuracy of clinical assessments in primary care settings and reducing the unnecessary utilization of healthcare resources.
